# Antiplatelet treatment in different clinical settings

**DOI:** 10.1093/ehjcvp/pvaf060

**Published:** 2025-08-28

**Authors:** Stefan Agewall

**Affiliations:** Institute of Clinical Sciences, Karolinska Institute of Danderyd, Stockholm Sweden

**Figure:**
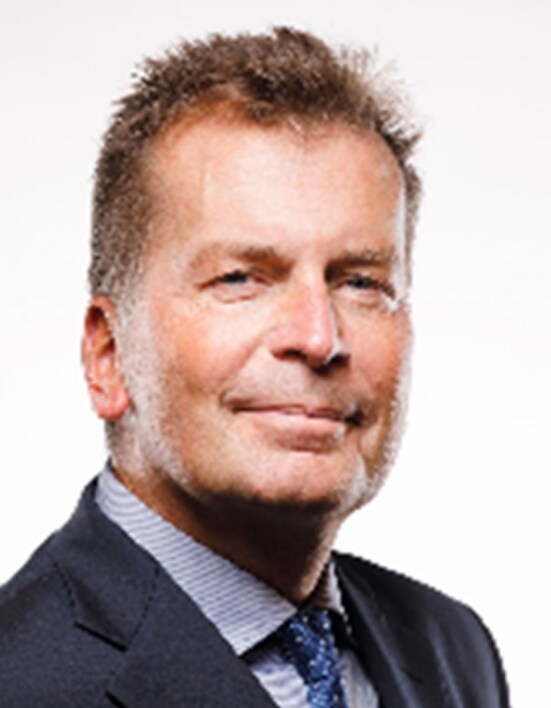


Cardiogenic shock (CS) is a serious complication of acute myocardial infarction (AMI) and remains an important cause of death with a rate of 30%–45%.^1,2^ While the question of pre-treatment with P2Y12 inhibitors has been well addressed in Non-ST Elevation Myocardial Infarction and ST Elevation Myocardial Infarction, there are currently sparse data in patients with AMI-CS. Dr Montalescot *et al*. from France, aimed to analyze the safety and effectiveness of a loading oral pre-treatment dose of P2Y12 inhibitor in patients with suspected AMI-CS. 421 patients with AMI-CS admitted to the catheterization laboratory within 24 h of admission in the ACTION-SHOCK cohort^3^ were included. The authors concluded that in patients with AMI-CS, pre-treatment with a P2Y12 inhibitor oral load was associated with an increased risk of major bleeding without benefit on major adverse cardiovascular events (MACE).

Approximately 30%–40% of patients hospitalized with Acute Coronary Syndrome (ACS) are 75 years or older,^4,5^ and old age is associated with increased risks of both ischaemic events and bleeding. Dr Marxer and co-workers from Sweden aimed to assess the risk of MACE and major bleeding in ACS patients ≥75 years (*n* = 4637) initiating ticagrelor vs. clopidogrel treatment by using healthcare data from the Stockholm region. After adjusting for 46 baseline confounders, they found that in ACS patients aged ≥75 years, ticagrelor was associated with a lower risk of MACE than clopidogrel. There were no differences in major bleeding.

There is a substantial variability in individual responses to antiplatelet therapy, which significantly influences the risk-benefit balance of these treatment.^6-10^ Dr Pulcinelli *et al*. from Italy analyzed 11 913 patients undergoing light transmission aggregometry using a standardized methodology to investigate the impact of sex on platelet reactivity with or without antiplatelet therapy. The study group concluded that females exhibit heightened baseline adenosine 5′-diphosphate (ADP)-dependent platelet reactivity and a diminished response to aspirin and clopidogrel monotherapy compared with males.

The optimal antithrombotic therapy to balance the risk of thrombosis and bleeding in patients who undergo transcatheter aortic valve implantation (TAVI) is unclear.^11,12^ In another paper from Italy, Dr Matti *et al*. aimed to evaluate and compare the outcomes of single antiplatelet therapy (SAPT) and dual antiplatelet therapy (DAPT) in patients with severe peripheral artery disease (PAD) and without atrial fibrillation (AF) undergoing TAVI. The study group used the HOSTILE registry^13^ which was a multicenter international study that collected data from 1707 patients with severe PAD undergoing TAVI. Among 573 patients without AF treated through transfemoral or non-thoracic alternative approach, 144 received SAPT and 429 DAPT after TAVI. DAPT was associated with reduced all-cause death at 12 months.

What is the best treatment for patients with AF who undergo percutaneous coronary intervention (PCI)^14,15^? Dr Choi and co-workers from Korea studied the efficacy and safety of aspirin vs. clopidogrel as a combination therapy with DOAC in a registry study with patient data from the Korea National Health Insurance Service (*n* = 9157). After propensity score, matching patients with AF receiving dual antithrombotic therapy after PCI, aspirin and clopidogrel showed similar efficacy and safety when used in combination with DOAC.

Pre-treatment of ACS patients with oral antiplatelet drugs before PCI has gradually been downgraded in recent guidelines. Factors such as vomiting, altered physiology, sedatives, mechanical ventilation, and therapeutic hypothermia may impair drug absorption, reducing the intended antiplatelet effect and increasing ischaemic risk. In these cases, intravenous antiplatelet strategies with ASA and cangrelor might guarantee adequate periprocedural platelet inhibition. In a review paper in this issue of the journal, Dr Zeymer *et al*., discuss the role of cangrelor in acute and high-risk PCI settings.

Glucagon-like peptide-1 receptor agonists (GLP-1 RAs) have dual benefits of glucose lowering and cardiovascular risk reduction, but heterogeneity exists across cardiovascular outcome trials.^16-20^ In a review and meta-analysis, Dr Gibson *et al*. from US report results from ten trials (67 769 patients; 34 536 receiving GLP-1 RAs). The authors concluded that GLP-1 RAs significantly reduce MACE, cardiovascular death, and all-cause mortality in type II diabetes patients. Higher baseline BMI and older age were associated with greater cardiovascular benefit.
